# Mitochondrial metabolism of sexual and asexual blood stages of the malaria parasite *Plasmodium falciparum*

**DOI:** 10.1186/1741-7007-11-67

**Published:** 2013-06-13

**Authors:** James I MacRae, Matthew WA Dixon, Megan K Dearnley, Hwa H Chua, Jennifer M Chambers, Shannon Kenny, Iveta Bottova, Leann Tilley, Malcolm J McConville

**Affiliations:** 1 Department of Biochemistry and Molecular Biology, Bio21 Molecular Science and Biotechnology Institute, 30 Flemington Road, Parkville, VIC, 3010, Australia; 2 ARC Centre of Excellence for Coherent X-ray Science, University of Melbourne, 30 Flemington Road, Parkville, VIC, 3010, Australia

**Keywords:** Malaria, Central carbon metabolism, TCA cycle, Metabolomics, Gametocyte

## Abstract

**Background:**

The carbon metabolism of the blood stages of *Plasmodium falciparum,* comprising rapidly dividing asexual stages and non-dividing gametocytes, is thought to be highly streamlined, with glycolysis providing most of the cellular ATP. However, these parasitic stages express all the enzymes needed for a canonical mitochondrial tricarboxylic acid (TCA) cycle, and it was recently proposed that they may catabolize glutamine via an atypical branched TCA cycle. Whether these stages catabolize glucose in the TCA cycle and what is the functional significance of mitochondrial metabolism remains unresolved.

**Results:**

We reassessed the central carbon metabolism of *P. falciparum* asexual and sexual blood stages, by metabolically labeling each stage with ^13^C-glucose and ^13^C-glutamine, and analyzing isotopic enrichment in key pathways using mass spectrometry. In contrast to previous findings, we found that carbon skeletons derived from both glucose and glutamine are catabolized in a canonical oxidative TCA cycle in both the asexual and sexual blood stages. Flux of glucose carbon skeletons into the TCA cycle is low in the asexual blood stages, with glutamine providing most of the carbon skeletons, but increases dramatically in the gametocyte stages. Increased glucose catabolism in the gametocyte TCA cycle was associated with increased glucose uptake, suggesting that the energy requirements of this stage are high. Significantly, whereas chemical inhibition of the TCA cycle had little effect on the growth or viability of asexual stages, inhibition of the gametocyte TCA cycle led to arrested development and death.

**Conclusions:**

Our metabolomics approach has allowed us to revise current models of *P. falciparum* carbon metabolism. In particular, we found that both asexual and sexual blood stages utilize a conventional TCA cycle to catabolize glucose and glutamine. Gametocyte differentiation is associated with a programmed remodeling of central carbon metabolism that may be required for parasite survival either before or after uptake by the mosquito vector. The increased sensitivity of gametocyte stages to TCA-cycle inhibitors provides a potential target for transmission-blocking drugs.

## Background

The human parasite *Plasmodium falciparum* is the major cause of disease and death from malaria [1]. Disease is associated with the development of asexual parasite stages that undergo repeated cycles of invasion and replication in red blood cells (RBCs). Following establishment of infection, a small proportion of parasites (<1%) differentiate to gametocytes [2,3]. Gametocytogenesis is essential for subsequent transmission because the mature gametocyte is the only stage that can undergo sexual development in the mosquito vector, which is a prerequisite for the spread of disease*.* Both the asexual RBC stages and gametocytes are thought to be primarily dependent on glucose uptake and glycolysis for ATP synthesis and survival. Glucose uptake in infected RBCs increases more than 75-fold compared with uninfected RBCs [4], and the resultant increased lactate production contributes to lactic acidosis, a major cause of morbidity and death during severe malaria [5]. Despite their reliance on glycolysis, the asexual stages of *P. falciparum *retain a single mitochondrion that is essential for parasite growth. Maintenance of the mitochondrial respiratory chain appears to be required for both the transport of proteins and metabolites into the mitochondrion and for the reoxidation of inner-membrane dehydrogenases, such as the dihydroorotate dehydrogenase involved in *de novo* pyrimidine biosynthesis [6]. As a result, the asexual stages and gametocytes are sensitive to electron transport chain inhibitors, including the antimalarial atovaquone [6-9].

Mitochondrial dehydrogenases require a source of reducing equivalents which could, in principal, be generated in the mitochondrion or in the cytoplasm. *P. falciparum* encodes all of the enzymes needed for a complete TCA cycle, but the genes encoding a mitochondrial pyruvate dehydrogenase (PDH) complex are missing [10], and it is generally assumed that a TCA cycle utilizing glycolytic pyruvate does not operate in the blood stages [11-16]. The possibility that an unusual branched TCA cycle may operate in the asexual stages of *P. falciparum*, fuelled by the catabolism of glutamine via both the oxidative and reductive arms of the TCA cycle, was recently proposed [13], but subsequently retracted [17]. More recent genetic studies have also suggested that operation of a complete mitochondrial TCA cycle is not required for the development of the asexual stages in the related murine parasite, *Plasmodium berghei*[18,19]. The extent to which a conventional or unconventional TCA cycle operates in *P. falciparum *RBC stages therefore remains unresolved.

Despite lacking a recognizable mitochondrial PDH in *P. falciparum*, there is increasing evidence that a conventional TCA cycle can operate in the insect stages of these parasites [18,20] and of other apicomplexan parasites. In particular, we have recently shown that the TCA cycle is essential for the growth of intracellular stages of *Toxoplasma gondii*[21].

In this study, we therefore reinvestigated the potential role of mitochondrial metabolism in *P. falciparum* asexual stages, and the possibility that the TCA cycle is important for the development of gametocytes.

## Results

**Figure 1:**
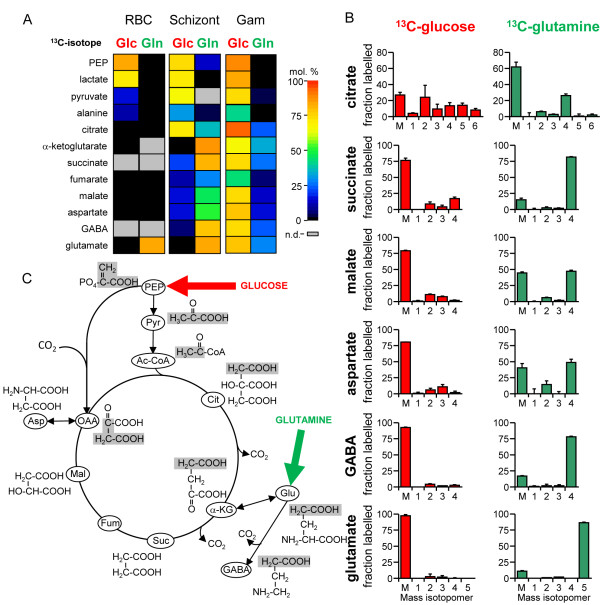
***Plasmodium falciparum *asexual and gametocyte stages catabolize glucose and glutamine in a canonical tricarboxylic acid (TCA) cycle.** Gametocytes, ring-stage parasite-infected, and uninfected red blood cells (RBCs) were suspended in medium containing either ^13^C-U-glucose or ^13^C-U-glutamine. (**A**) Metabolites were extracted from schizont-infected and uninfected RBCs (at 38 hours) and gametocytes (at 24 hours), and incorporation of ^13^C into polar metabolites was quantified by gas chromatography–mass spectrometry (GC-MS). Heat plots show enrichment (mol% containing one or more ^13^C carbons) after correction for natural abundance (n = 3 to 4). (**B**) Abundance of TCA-cycle isotopomers in schizont-infected RBCs. The *x*-axis indicates the number of ^13^C atoms in each metabolite (‘M’ indicates the monoisotopic mass containing no ^13^C atoms). Error bars indicate SD (n = 3 to 4). (**C**) Labeling of intermediates via the TCA cycle inferred from the isotopomer analysis. Grey boxes indicate fate of carbons in phosphoenolpyruvate (PEP) in indicated TCA-cycle intermediates. Both ^13^C_2_-acetyl-CoA and ^13^C_3_-oxaloacetic acid can be generated from ^13^C_3_-PEP, leading to formation of +2, +3, and +5 citrate. Uniformly labeled citrate can be generated through multiple rounds through the TCA cycle. Glutamine can enter the TCA cycle after its catabolism to α-ketoglutarate. Abbreviations: α-KG, α-ketoglutarate; Ac-CoA, acetyl-CoA; Asp, aspartate. Cit, citrate; Fum, fumarate; GABA, γ-aminobutyric acid; Glu, glutamate; Mal, malate; n.d., not detected; OAA, oxaloacetate; Pyr, pyruvate; Suc, succinate.

Uninfected RBCs and synchronized, ring stage-infected RBCs were metabolically labeled with ^13^C-U-glucose or ^13^C-U-glutamine for 38 hours, and then rapidly chilled. This was followed by extraction of intracellular metabolites and quantification of ^13^C-enrichment by gas chromatography–mass spectrometry (GC-MS) (see Additional file 1). Incubation of uninfected and infected RBCs in medium containing ^13^C-glucose led to a high level of enrichment (>75%) in glycolytic intermediates, including phosphoenolpyruvate (PEP) and lactate (Figure 1A). Despite the parasite lacking a recognizable mitochondrial isoform of pyruvate dehydrogenase, labeling of citrate and a range of other TCA-cycle intermediates also occurred in *P. falciparum*-infected RBCs under these conditions. Although TCA-cycle intermediates were detected in uninfected RBCs, they were present at concentrations more than five-fold lower than those of infected RBCs, and labeling of these intermediates was negligible (Figure 1A). The predominant isotopomers of citrate in ^13^C-glucose-fed infected RBC contained +2, +4 or +6 labeled carbons, indicating the operation of a canonical TCA cycle in which pyruvate feeds into the cycle via acetyl-CoA (Figure 1B,C). Citrate isotopomers containing +3 and +5 labeled carbons were also detected, reflecting the activity or activities of the *Plasmodium* PEP carboxylase (PEPC) and/or PEP carboxykinase (PEPCK) that catalyze the carboxylation of ^13^C_3_-phosphoenolpyruvate (PEP) to ^13^C_3_-oxaloacetate. Isotopomer analysis of other intermediates in the TCA cycle provided further support for this model (Figure 1B). However, the cellular pools of these intermediates were labeled to a much lower extent than occurred with citrate, indicating entry of other unlabeled carbon sources into the TCA cycle (see below) (Figure 1B). Significant levels of labeled γ-aminobutyric acid (GABA) were also detected in infected, but not in uninfected RBCs (Figure 1A). Labeling of GABA provides evidence for the presence of a partial GABA shunt in which intermediates from the TCA cycle are used to synthesize glutamate, which is subsequently decarboxylated to GABA, as has recently been shown to occur in *T. gondii*[21].

The operation of a conventional TCA cycle in the *P. falciparum* asexual stages was confirmed by complementary ^13^C-glutamine labeling experiments. Incubation of infected RBC with ^13^C-glutamine resulted in labeling of all detectable TCA-cycle intermediates, with the highest ^13^C-enrichment seen in α-ketoglutarate and C4 dicarboxylic acids (Figure 1A). No labeling of TCA-cycle intermediates was detected in uninfected RBCs (Figure 1A). The predominant isotopomers of succinate, malate, and fumarate in ^13^C-glutamine-labeled parasites were fully labeled, indicating that most of the carbon skeletons that enter the TCA cycle via glutamate are not continuously cycled through the TCA reactions, and are presumably exported from the mitochondrion. This was supported by the low level of labeling of citrate compared with the C4 dicarboxylic acids and the predominant presence of the +4 citrate isotopomer. However, all intermediates contained readily detectable levels of +2 isotopomers, consistent with cycling of a sub-pool of C4 dicarboxylic acids around a canonical oxidative TCA cycle. Importantly, and in contrast to a previous report [13], citrate isotopomers containing +5 labeled carbons were not detected, indicating minimal catabolism of α-ketoglutarate via the reductive arm of the TCA cycle (Figure 1B). Collectively, these analyzes show that the *P. falciparum* asexual stages catabolize both pyruvate and glutamate in a conventional TCA cycle, and argue against the operation of a bifurcated TCA cycle, as previously proposed [13,17]. However, these results do support a degree of compartmentalization within this cycle, with glutamate sustaining a major flux from α-ketoglutarate to malate/oxaloacetate, and with glucose-derived pyruvate and oxaloacetate contributing to a minor flux towards citrate synthesis.

**Figure 2:**
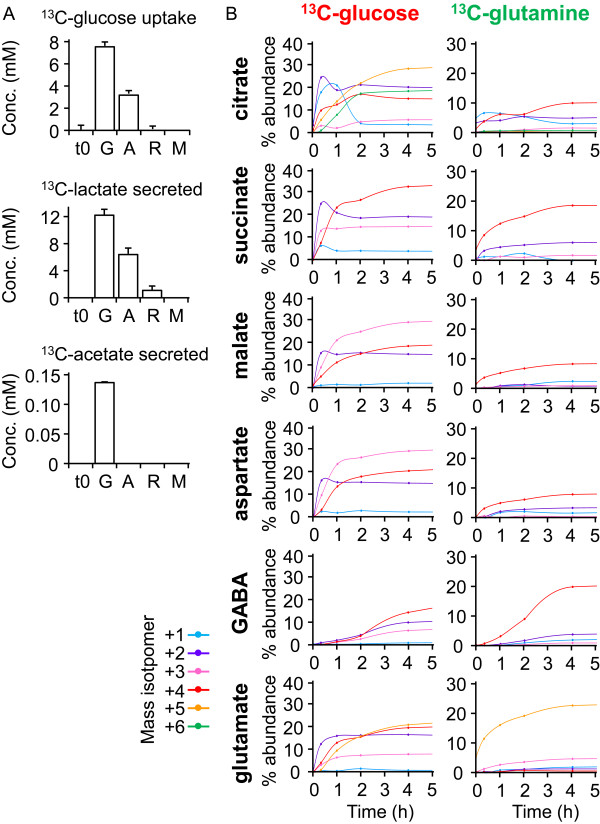
***Plasmodium falciparum *gametocytes primarily use glucose to fuel the tricarboxylic acid (TCA) cycle.** (**A**,**B**)Trophozoite-infected red blood cell (RBCs) (A) and gametocytes (G), and uninfected RBCs (R) were cultivated in medium containing 8 mmol/l^13^C-U-glucose or ^13^C-U-glutamine. (**A**) Uptake of ^13^C-glucose and secretion of ^13^C-lactate and ^13^C-acetate was monitored by ^13^C nuclear magnetic resonance imaging (^13^C NMR) analysis of the medium over 24 hours, and compared with the initial medium (t0). No changes were seen in glucose, lactate, or acetate levels in medium lacking RBCs (M). (**B**) Gametocytes were cultured in the presence of ^13^C-glucose or ^13^C-glutamine for the indicated times, and ^13^C-enrichment in key metabolites determined by gas chromatography–mass spectrometry (GC-MS). Changes in the major isotopomer of each metabolite over the first 5 hours are shown. Results are the mean of three biological replicates.

A small proportion of asexual parasite stages differentiate to gametocytes *in vivo,* providing a pool of transmission-competent parasites. Development of *P. falciparum* gametocytes involves distinct morphological transitions (stages I to V) and takes 7 to 10 days to complete [22]. During early-stage gametocytogenesis, newly invaded parasites expand in size, with concomitant depletion of host-cell cytoplasm and hemoglobin [23,24]. These parasite stages are non-replicating, and are thought to enter a metabolically quiescent state by Stage III as they become less insensitive to current first-line anti-malarial drugs [25]. To measure the metabolic state of developing gametocytes more precisely, ^13^C-glucose uptake by Stage III gametocyte-infected RBCs was monitored by ^13^C-nuclear magnetic resonance spectroscopy (NMR). Unexpectedly, gametocyte-infected RBCs exhibited a significantly higher rate of ^13^C-glucose utilization than did RBCs infected with mature asexual stages, which was matched by increased rates of ^13^C-lactic acid secretion (Figure 2A; see Additional file 2). Gametocytes also produced significant amounts of ^13^C-acetate, indicating increased conversion of glucose into acetyl-CoA synthesis via either mitochondrial or apicoplast pathways.

To further define the metabolic state of gametocytes, Stage III gametocytes were cultured in the presence of ^13^C-glucose or ^13^C-glutamine, and enrichment in intracellular intermediates was determined by GC-MS. Marked differences were seen in the relative level of labeling of the TCA-cycle intermediates in gametocyte stages compared with the asexual stage parasites (Figure 1A). Specifically, TCA-cycle intermediates in gametocytes were strongly labeled with ^13^C-glucose, whereas the levels of enrichment in ^13^C-glutamine-fed gametocytes were low. The rapid labeling of citrate isotopomers with +2, +4, and eventually +6 labeled carbons after 2–4 hours demonstrates the operation of an active canonical TCA cycle (Figure 2B). Similarly, the rapid labeling of +3 isotopomers of oxaloacetate (indicated by aspartate labeling) and malate and the +5 isotopomer of citrate indicated that phosphoenolpyruvate may feed directly into the TCA cycle via PEP carboxylase or PEP carboxykinase (Figure 2B). Although labeling of gametocyte TCA-cycle intermediates with ^13^C-glutamine was relatively low compared with that in ^13^C-glucose-fed gametocytes, isotopomer analysis again confirmed conventional TCA-cycle operation. Specifically, a major +4 isotopomer was detected in all TCA intermediates, and this reached a maximum after 4 hours (Figure 2B). In contrast, +2 isotopomers were generated with slower kinetics, indicative of loss of labeled carbon with repeated cycles around the TCA cycle. As for the asexual stages, the absence of a predominant +5 isotopomer in citrate confirmed that the TCA cycle operates primarily in the oxidative direction in *P. falciparum* gametocytes (Figure 2B). GABA was labeled with both ^13^C-U-glucose and ^13^C-U-glutamine to the same maximal labeling level as other metabolites, although with slower kinetics (Figure 2B). Together, these findings suggest that there is substantial remodeling of mitochondrial metabolism in gametocytes, with glucose providing most of the carbon skeletons for operation of a complete TCA cycle (Figure 1A). Quantification of the rates of glucose consumption and lactate production provided further support for increased flux of glucose-derived pyruvate into the TCA cycle of gametocytes. Specifically, whereas more than 93% of the glucose internalized by the asexual stages was secreted as lactate, this was reduced to 80% in gametocytes, reflecting increased mitochondrial catabolism (Figure 2A).

**Figure 3:**
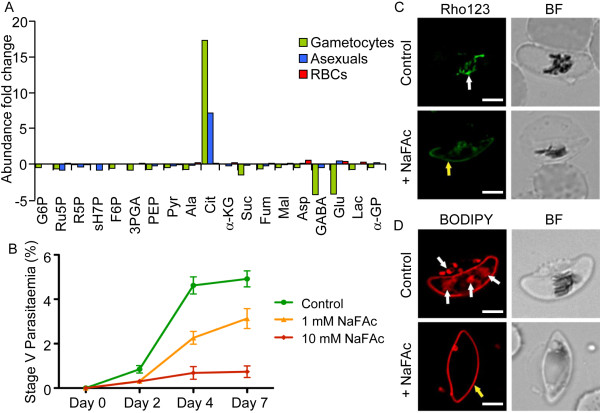
***P. falciparum *gametocytes exhibit increased sensitivity to mitochondrial tricarboxylic acid (TCA)-cycle inhibitors.** Ring-infected red blood cells (RBCs), gametocytes, and uninfected RBCs were cultured in the presence or absence of sodium fluoroacetate (NaFAc), and the metabolic and morphological effects were assessed. (**A**) After culturing in the presence or absence of 1 mmol/l NaFAc, metabolites were extracted from schizont-infected and uninfected RBCs (at 38 hours) and gametocytes (at 24 hours) and analyzed by gas chromatography–mass spectrometry (GC-MS). Bars represent abundance of metabolites in drug-treated cells compared with a drug-free control. Results are the mean of three to four biological replicates. Abbreviations: 3PGA, 3-phosphoglycerate; α-GP, α-glycerophosphate; α-KG, α-ketoglutarate; Ala, alanine; Asp, aspartate. Cit, citrate; F6P, fructose 6-phosphate; Fum, fumarate; G6P, glucose 6-phosphate; GABA, γ-aminobutyric acid; Glu, glutamate; Lac, lactic acid; Mal, malate; Pyr, pyruvate; R5P, ribose 5-phosphate; Ru5P, ribulose 5-phosphate; sH7P, *sedo*-heptulose 7-phosphate; Suc, succinate, (**B**) Gametocytes were cultured in standard culture medium with or without the addition of 1 or 10 mmol/l NaFAc. Stage distribution and parasitemia levels were assessed in smears made on days 0 to 7, and the percentage of fully mature (Stage V) gametocytes calculated (see Additional file 6 for representative smears). Error bars represent SEM, where n = 3. Day 7 gametocytes were labeled with (**C**) rhodamine-123 (Rho123) or (**D**) BODIPY-TR-ceramide. In untreated gametocytes, these dyes accumulated (white arrows) in (**C**) the reticulate mitochondrion and (**D**) intracellular membranes, but redistributed to the parasite plasma membrane in treated parasites (yellow arrows). Bright field (BF) images are shown. Scale bar = 3 μm.

Sodium fluoroacetate (NaFAc) is a selective and potent inhibitor of the TCA-cycle enzyme aconitase [26]. Metabolite profiling of asexual and gametocyte cultures treated with 1 mmol NaFAc revealed a 7-fold and 17-fold accumulation of citrate, respectively, with a concomitant decrease in abundance of downstream TCA metabolites (Figure 3A; see Additional file 3), supporting specific inhibition of the aconitase reaction in an oxidative cycle. Interestingly, levels of glutamate and GABA were also reduced (Figure 3A; see Additional file 3), possibly reflecting increased glutaminolysis and utilization of intracellular pools of these amino acids. To examine whether perturbation of the TCA cycle affects growth of the asexual stages or gametocyte development, asexual stage parasites or Stage II/III gametocytes were cultured in the presence of 1 mmol or 10 mmol NaFAc or sodium acetate (NaAc). No significant effect on the growth of asexual stages was seen after 7 days of continuous culture in the presence of NaFAc (see Additional file 4). The resistance of this stage to NaFAc is consistent with the predominant flux in the TCA cycle being from α-ketoglutarate to malate, downstream of the aconitase reaction. By contrast, gametocyte maturation was markedly reduced in the presence of NaFAc (Figure 3B). Under controlled conditions, most Stage II/III gametocytes progressed to Stage IV by day 2, and to Stage V by day 4 of the assay, with a gradual decrease in parasitemia (see Additional files 5 and 6) [27]. Treatment with NaFAc resulted in a dramatic, dose-dependent decrease in the development of viable Stage V gametocytes (Figure 3B; see Additional files 5 and 6). Treatment of gametocytes with 10 mmol NaFAc was associated with the loss of the mitochondrial membrane potential, as shown by the absence of reticular rhodamine-123 staining (Figure 3C) [28]. This was also associated with reduced labeling of intracellular membranes with a red fluorescent dye (BODIPY-TR-ceramide; Invitrogen) (Figure 3D), reflecting decreased uptake and/or integrity of intracellular organelles [29,30]. Thus, disruption of the TCA cycle in gametocytes may have pleiotropic effects on multiple processes, leading to loss of viability.

## Discussion

**Figure 4:**
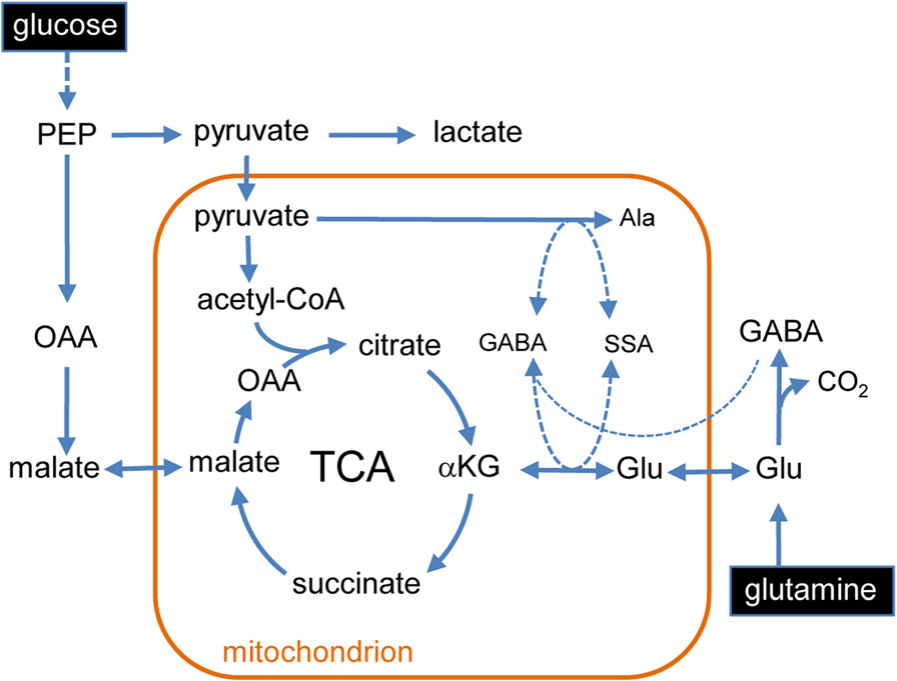
**Proposed model of glucose and glutamine metabolism in *Plasmodium falciparum *asexual and gametocyte stages.***P. falciparum* asexual and gametocyte stages catabolize host glucose and glutamine in mitochondria via the tricarboxylic acid (TCA) cycle. The major carbon fluxes around the TCA cycle in the asexual stages are driven by input of carbon skeletons derived from glutamine. In gametocytes, carbon skeletons derived from glucose (pyruvate, oxaloacetic acid) drive the major flux. Label incorporation from ^13^C-glutamine suggested that γ-aminobutyric acid (GABA) synthesized from exogenous and endogenous glutamate may participate in transamination reactions in the mitochondrion (dotted lines). Abbreviations: αKG, α-ketoglutarate; Ala, alanine; GABA, γ-aminobutyric acid; Glu, glutamate; OAA, oxaloacetate; SSA, succinic semi-aldehyde.

Our data allow a major revision of current models of central carbon metabolism of *P. falciparum* RBC stages by showing that both asexual and sexual stages utilize a canonical oxidative mitochondrial TCA cycle to catabolize host glucose and glutamine (Figure 4). Carbon skeletons derived from either glucose or glutamine enter the TCA cycle via acetyl-CoA or anaplerotic reactions, or α-ketoglutarate, respectively. Significantly, we found no evidence for operation of a bifurcated or branched TCA cycle in which glutamine is converted to malate via both oxidative and reductive ‘arms’ of the TCA cycle, as previously proposed [13,17]. *P. falciparum* asexual RBC stages seem to have a compartmentalized TCA cycle, in which carbon backbones derived from glucose sustain a minor flux from oxaloacetic acid to citrate, whereas carbon backbones derived from glutamine are used to sustain a higher flux from α-ketoglutarate to malate. Based on the rate of glucose consumption and glycolysis (lactate production), less than 7% of the internalized glucose is catabolized in the mitochondrion. A low flux of glucose and glutamate into the TCA cycle may be required for the generation of reducing equivalents for the essential respiratory chain and synthesis of succinyl-CoA for heme biosynthesis [12]. However, as shown here, chemical inhibition of the entry of glucose-derived intermediates into the TCA cycle had no detectable effect on the development of asexual stages, possibly reflecting continued operation of glutaminolysis and catabolism of the carbon backbones of glutamate in this cycle.

*P. falciparum* and other apicomplexan parasites lack a mitochondrial isoform of PDH, and the enzyme involved in converting pyruvate to acetyl-CoA remains to be characterized. A possible candidate for this function is the mitochondrion-located branched chain α-keto acid dehydrogenase (BCKDH) complex [31]. This complex is retained in all apicomplexa (except *Cryptosporidium* spp.), as well as more distantly related protists that lack a mitochondrial PDH [32], and has been shown to utilize pyruvate in some organisms [33,34]. The transcriptional upregulation of key subunits in the BCKDH complex (PF3D7_1312600) in *P. falciparum* gametocytes [35], may underlie the increased flux of pyruvate into the TCA cycle seen in this stage. However, entry of glutamine/glutamate into the *P. falciparum* TCA cycle could be regulated by either the glutamate dehydrogenase or by amino acid transaminases [36,37]. Because the *P. falciparum* glutamate dehydrogenase is not required for growth of asexual stages [37], it is likely that amino acid transaminases alone can fulfill this role. We have previously shown that *P. falciparum* encodes a putative glutamate/GABA transaminase (PF3D7_0608800), as well as the proteins needed for synthesis of GABA (PF3D7_0405700) and import of this metabolite into the mitochondrion (PF3D7_0209600) [21]. The finding that GABA is synthesized in both asexual and sexual RBC stages, and that pyruvate is converted to alanine is consistent with both metabolites contributing to transamination reactions in the mitochondrion, which convert glutamate to α-ketoglutarate (Figure 4) [38]. It is notable that *P. falciparum* lacks an obvious homolog for succinate semi-aldehyde dehydrogenase, which is required for the conversion of GABA to the TCA-cycle intermediate succinate [21]. This differs from the situation in the related apicomplexan parasite, *Toxoplasma gondii*, which is able to utilize GABA as a short-term energy reserve [21], highlighting further specializations in the carbon metabolism of the Apicomplexa.

Despite entering a non-proliferative state, gametocytes exhibit increased levels of glucose utilization, glycolytic flux, and TCA catabolism of pyruvate. This switch to a more efficient method of energy generation may be needed to sustain gametocyte development under conditions of hypoglycemia, which is common in patients with severe malaria [39,40]. The upregulation of TCA function in gametocytes might also reflect increased energy demands in female gametes preparing for the post-fertilization stages, during which access to glucose in the mosquito hemolymph may be limited [41]. Indeed, there is accumulating evidence that a functional TCA cycle is essential for the development of the ookinete [18]. Interestingly, deletion of the TCA-cycle enzyme succinate dehydrogenase had no observable effect on the production of *P. berghei* gametocytes [18]. By contrast, we found that partial inhibition of the TCA cycle with NaFAc inhibits gametocyte development, and high concentrations of NaFAc are lethal to this stage. These apparent differences could reflect species-specific differences in their dependence on the TCA for energy or maintenance of the respiratory chain, or the possibility that inhibition of the aconitase reaction results in a stronger metabolic phenotype, including reduced yield of NADH and/or the accumulation of intermediates such as citrate to toxic levels. Further gene knockout studies are required to definitively validate these possibilities.

The metabolic switch to increased TCA-cycle catabolism of glucose in gametocytes could also reflect changes in carbon-source availability in the infected RBC. Glutamine uptake in trophozoite-infected RBC is mediated by a specific RBC amino acid transporter [42] and novel permeation pathways (NPPs) induced by the parasite [43]. There is evidence that the NPPs are downregulated in developing gametocytes [44], resulting in reduced glutamine uptake. The reduced availability of glutamine in gametocytes might explain the switch to glucose as the preferred carbon source for TCA metabolism.

Recent transcriptional studies have suggested the existence of at least two physiological states of blood stage parasites in the human host, including a glycolysis-dependent asexual state and a ‘weakly gametocyte/sporozoite-like’ state that seems to have upregulated non-glycolytic energy metabolism, including the TCA cycle and oxidative phosphorylation [40,45,46]. Our results add to these findings, and suggest that the transcriptional upregulation of TCA cycle enzymes in response to glucose limitation or other metabolic stresses possibly represents a switch to a more energy-efficient metabolism.

## Conclusion

Our findings reveal major changes in metabolic fluxes in *P. falciparum* bloodstream stages, which are not reflected in transcriptional-profiling studies, highlighting the importance of post-transcriptional regulatory mechanisms in controlling parasite metabolism. Stage-specific changes in metabolic fluxes in core metabolic pathways may be exploited in drug development. In particular, our results suggest that inhibitors of the mitochondrial enzyme responsible for converting pyruvate to acetyl-CoA could lead to a defect in gametocytogenesis. Field studies and mathematical modeling show that such transmission-blocking inhibitors will be needed to achieve the long-term goal of malaria elimination [47,48].

## Methods

### Parasite culture

*P. falciparum* asexual and gametocyte cultures were prepared as described previously [49]. Briefly, asexual stage *P. falciparum* 3D7 parasite-infected RBCs were cultured in O-positive RBCs (Australian Red Cross Blood Service (ARCBS), Carlton, VIC, Australia) at 4 to 5% hematocrit, in a commercial medium (RPMI-GlutaMAX^(^™^)^-HEPES; Invitrogen) supplemented with 5% v/v human serum (ARCBS), and 0.25% w/v lipid-rich bovine serum albumin (AlbuMAX II; Invitrogen). Sorbitol synchronizations were used to obtain ring-stage cultures. Gametocyte cultures were initiated at 2% trophozoites (5% hematocrit) and grown until they reached 8 to 10% trophozoites. Volumes of parasite cultures were expanded four times, resulting in a 2% trophozoite stage parasite culture (day 0). Gametocytes were grown in the presence of 62.5 mmol/l N-acetylglucosamine from day 1 of culture. Development of the cultures was monitored by Giemsa-stained thin smears. Stage III (day 5) and Stage V (day 11) gametocytes were enriched by magnetic separation as previously described [49]. For inhibitor studies, the culture medium was supplemented with either 1or 10 mmol NaFAc or NaAc. The concentration of these supplements was maintained during subsequent medium changes. All cell lines used in this study were certified to be free of *Mycoplasma* contamination by PCR of culture-derived DNA using *Mycoplasma*-specific primers (WEHI Antibody Facility, Bundoora, Victoria, Australia).

### Stable isotope labeling and metabolite extraction of *P. falciparum*-infected and uninfected RBCs

Stable isotope labeling and metabolite extraction was adapted from that previously described [21]. Synchronized *P. falciparum*-infected cultures (at 8 to 10% parasitemia) and uninfected RBC were pelleted (800 × *g*, 10 min, 4°C) and the medium replaced with glucose-free RPMI medium (Sigma-Aldrich, St Louis, MO, USA) or glutamine-free RPMI media (Invitrogen), supplemented as above with an additional 8 mmol/l final concentration of ^13^C-U-glucose or ^13^C-U-glutamine (Spectra Stable Isotopes, Columbia, MD, USA), as indicated. At required time points, cultures were rapidly transferred to a 50 ml centrifuge tube, and cellular metabolism was quenched by immersing the tube in a dry ice/ethanol slurry to chill the suspension to 0°C [21]. Schizont-infected RBCs were purified from uninfected and ring-infected RBCs by passage through a size LD column and magnetic unit apparatus (varioMACS; Miltenyi Biotec, Bergisch Gladbach, Germany) [49], at 4°C. Mature trophozoite-infected and schizont-infected RBCs were eluted with ice-cold PBS at one-fifth of the culture volume. Gametocyte cultures were magnet-purified at the desired stage of development 1 day prior to commencement of the labeling experiments. Infected and uninfected RBCs were pelleted by centrifugation (800 × *g* for 10 minutes at 4°C), and washed three times with ice-cold PBS. Aliquots of 10^8^ cell equivalents were extracted with chloroform:methanol (2:1 v/v) for 1 hour at 4°C with periodic sonication. The samples were separated by centrifugation (18,000 × *g* for 10 minutes at 4°C), the supernatant retained, and the pellet re-extracted with methanol:water (2:1 v/v containing 1 nmol *scyllo*-inositol as internal standard) for 1 hour, as above. After centrifugation, the supernatants were pooled and dried under nitrogen. Polar and apolar metabolites were separated by phase partitioning (chloroform:methanol:water, 1:3:3 v/v). Polar metabolite extracts were dried in a rotary evaporator, washed twice with methanol, derivitized by methoximation and trimethylsilylation, and analyzed by GC-MS [26]. The level of labeling of individual metabolites was estimated as the percentage of the metabolite pool containing one or more ^13^C atoms after correction for natural abundance. The mass isotopomer distributions of individual metabolites were corrected for the occurrence of natural isotopes in both the metabolite and the derivitization reagent [50]. To ensure that the starting medium was consistent between experiments, aliquots (10 μl) were washed, derivitized, and analyzed by GC-MS (as above) with each experiment.

### Analysis of *P. falciparum*-infected and uninfected RBC culture medium

Synchronized *P. falciparum*-infected cultures and uninfected RBCs (2 × 10^9^ cells) were cultured in 10 ml glucose-free medium supplemented as above, with 8 mmol ^13^C-U-glucose and 8 mmol ^12^C-U-glutamine. Both early trophozoite-infected and Stage III gametocyte-infected cultures were at 10% infection. At required time points, 2 × 600 μl aliquots were removed and separated by centrifugation (18,000 × *g* at room temperature for 1 minute) to remove RBCs. The volume of culture remaining at each time point was measured to account for evaporative losses. Culture supernatants (540 μl) were gently pre-mixed with 5 mmol D6-DSS in deuterium oxide (D_2_O) (60.0 μl, containing 0.2% w/v NaN_3_) and 21.4 mmol ^13^C-U-glycerol in D_2_O (5.00 μl, containing 0.2% w/v NaN_3_), prior to analysis by NMR. ^13^C spectra at 200 MHz were obtained using an 800 MHz NMR spectroscope (Avance; Bruker-Biospin, Rheinstetten, Germany) fitted with a cryoprobe. Samples were maintained at 25°C and spun at 20 Hz during sample collection. ^13^C spectra were acquired using the Avance zgpg pulse program with power-gated ^1^H decoupling. A pre-scan delay of 80.78 μ seconds, a delay between pulses of 2.0 seconds, and an acquisition time of 0.78 seconds were used. For each sample, four dummy scans were followed by 4000 scans with receiver gain set to 2050. The resulting ^13^C free induction decays were processed with Bruker TOPSPIN version 2.0 (the exponential function with line broadening = 5.0 Hz was applied in the frequency domain prior to Fourier transformation, baseline correction, and integration). Metabolite abundances were quantified as described previously [51] by multiplication of the metabolite integration area(s) with a correction factor derived from five T1 relaxation NMR experiments of known metabolite concentrations and normalization to the internal standard (^13^C-glycerol).

### Gametocyte morphology analysis

Purified Stage II to III gametocytes were used to initiate 10 to 15% parasitemia cultures at 1% hematocrit. Treatment groups included complete culture media with or without NaFAc (10 mmol/l) or sodium acetate (10 mmol/l), and were performed in duplicate and on two separate occasions. Culture media and drug were exchanged daily. Development of gametocytes were monitored daily by Giemsa-stained slides, and percentage parasitemia was calculated from the counts of approximately 20 random fields of view (approximately 2000 uninfected RBCs) from each slide, and mean values and standard errors were estimated. Gametocyte morphology was classified as previously described [49].

### Fluorescence labeling

Membrane organization within the gametocytes was assessed by staining (BODIPY-TR-ceramide; Invitrogen Corp. Carlsbad, CA, USA). Parasites were incubated overnight in the presence of BODIPY-TR-ceramide in complete culture medium at a final concentration of 0.7 μmol/l, as previously described [48]. Mitochondrial membrane potential was investigated using the membrane potential dye Rho123 (Invitrogen Corp. Carlsbad, CA, USA). Staining was performed essentially as described previously [28]. Briefly, gametocytes were resuspended in 0.1 μg/ml Rho123 in complete culture media, and incubated for 30 minutes at 37°C. The cells were pelleted and resuspended in normal culture medium, and incubated for an additional 30 minutes at 37°C. Cells were imaged using a microscopy system (DeltaVision Elite; Applied Precision, Issaquah, WA, USA). Images were deconvolved using the default settings in the softWoRx acquisition software (version 5.0). Images were further processed using NIH ImageJ (version 1.47c; [52]).

## Abbreviations

α-KG: α-ketoglutarate; BCKDH: Branched chain α-keto acid dehydrogenase; BF: Brightfield images; D2O: Deuterium oxide; EIC: Extracted ion chromatogram; GABA: γ-Aminobutyric acid; GC-MS: Gas chromatography–mass spectrometry; Glu: Glutamate; Mal: Malate; NaAc: Sodium acetate; NaFAc: Sodium fluoroacetate; NMR: Nuclear magnetic resonance spectroscopy; NPP: Novel permeation pathway; OAA: Oxaloacetate; PBS: Phosphate-buffered saline; PDH: Pyruvate dehydrogenase; PEP: Phosphoenolpyruvate; PEPC: Phosphoenolpyruvate carboxylase; PEPCK: Phosphoenolpyruvate carboxykinase; Pyr: Pyruvate; RBC: Red blood cell; Suc: Succinate; TCA: Tricarboxylic acid; TIC: Total ion chromatogram.

## Competing interests

The authors declare that they have no competing interest.

## Authors’ contributions

JIM, MWAD, LT and MJM conceived of the study. LT and MJM participated in the design and coordination of the study. JIM undertook the metabolomic analyses. MWAD undertook the gametocyte analyses. MKD, SK, and IB contributed to the gametocyte studies. HHC and JMC contributed to the metabolomics studies. JIM, MWAD, LT, and MJM helped to draft the manuscript. All authors read and approved the final manuscript.

## Supplementary Material

Additional file 1 **Gas chromatography–mass spectrometry (GC-MS) chromatograms of *Plasmodium falciparum*-infected and uninfected red blood cell (RBC) polar metabolites.** Gametocytes (top row), schizont-infected RBCs (asexuals, middle row), and uninfected RBCs (bottom row) were harvested, and metabolites extracted as described. The panels depict representative total ion chromatograms (TICs) and extracted ion chromatograms (EICs) of metabolites extracted from 10^8^ cells. Panels in the left-hand column show full TICs. The area of the chromatogram containing the tricarboxylic acid (TCA) metabolites (purple box) is shown in detail in the panels of the middle column. Quantification was performed as described, using extracted ions to distinguish between overlapping peaks. An example is shown in the EIC panels in the right-hand column, depicting the area highlighted by the orange box in the TICs. Lines represent the monoisotopic (diagnostic) ion for aspartic acid (green), glutamine or glutamic acid (blue), and γ-aminobutyric acid (GABA; red) (note the difference in the *y*-axes). Abundances are shown in arbitrary units, with absolute quantifications shown in Additional file 3. NB: Quantification for glutamic acid was performed using the peak (E) corresponding to glutamate alone. Abbreviations: C, citrate; D, aspartate; E, glutamate; F, fumarate; G, GABA; M, malate; S, succinate; X (Gln/Glu), glutamine/glutamate. Click here for file

Additional file 2 ^**13**^**C Nuclear magnetic resonance spectroscopy (NMR) spectra of *Plasmodium falciparum*-infected and uninfected red blood cell (RBC) culture medium.** Early trophozoite-infected RBCs (A), gametocytes (G), and uninfected RBCs (R) were cultivated in medium containing 8 mmol/l ^13^C-U-glucose. Uptake of ^13^C-glucose, and secretion of ^13^C-lactate and ^13^C-acetate was monitored by ^13^C-NMR analysis of the medium over 24 hours and compared with the initial medium (t0) and medium lacking RBCs at 24 hours (M). Representative spectra, with peaks corresponding to glucose, lactate, and acetate, are shown. ‘L’ represents the second lactate peak cluster, and a magnified area corresponding to acetate (inset) is shown. Abundances are shown in arbitrary units on an equivalent *y*-axis, with absolute quantification shown in Figure 2A. Click here for file

Additional file 3 **Quantification of polar metabolites of *Plasmodium falciparum*-infected and uninfected red blood cells (RBCs) (related to Figure** 3**A).** Ring-infected RBCs, gametocytes, and uninfected RBCs were cultured in the presence or absence of NaFAc, and the metabolic and morphological effects were assessed. (A) After culturing in the presence or absence of 1 mmol sodium fluoracetate (NaFAc), metabolites were extracted from schizont-infected and uninfected RBCs (at 38 hours) and gametocytes (at 24 hours), and polar metabolites of interest were quantified by gas chromatography–mass spectrometry (GC-MS) and comparison with known standards. Abundances are shown in nmoles per 10^8^ cells, with numbers in brackets representing standard deviation, where n = 4 for gametocyte analyses, and n = 3 for asexual stage analyses. The abundance ratio of cells cultured in the presence of NaFAc to the absence of NaFAc is also shown. All values are to 2 d.p. Abbreviations are described in Figures 1 and 3. Click here for file

Additional file 4 **Growth effects upon treatment of asexual parasites with sodium fluoroacetate(NaFAc).** Asexual stage-infected red blood cells (RBCs) were cultured in standard culture medium (normal), with or without the addition of 1 or 10 mmol/l NaFAc or NaAc, with equivalent dilutions made to each of the cultures. Parasitemia levels were assessed in smears made on day 7. Results are from n = 3 biological replicates. Click here for file

Additional file 5 **Growth effects upon treatment of gametocytes with sodium fluoroacetate (NaFAc).** A Stage II/III culture of gametocytes was incubated under standard culture conditions with or without the addition of 1 or 10 mmol/l NaFAc or sodium acetate (NaAc). Stage distribution and parasitemia levels were assessed in Giemsa smears made on days 0 to 7 (see Additional file 6). Red, Stage II/III; blue, Stage IV; green, Stage V. Error bars represent SEM where n = 3 biological replicates. Click here for file

Additional file 6 **Giemsa-stained smears of gametocytes with sodium fluoroacetate (NaFAc).** A Stage II/III culture of gametocytes was incubated under standard culture conditions with or without the addition of 1 or 10 mmol/l NaFAc or sodium acetate (NaAc). Representative images of smears made on day 7 are presented. Click here for file
