# Supplementary figures and images for: Mitochondrial metabolism of sexual and asexual blood stages of the malaria parasite Plasmodium falciparum
Source: BMC Biol. 2013 Jun 13;11:67. doi: 10.1186/1741-7007-11-67 (PMC3704724; doi:10.1186/1741-7007-11-67)

# GAMETOCYTES

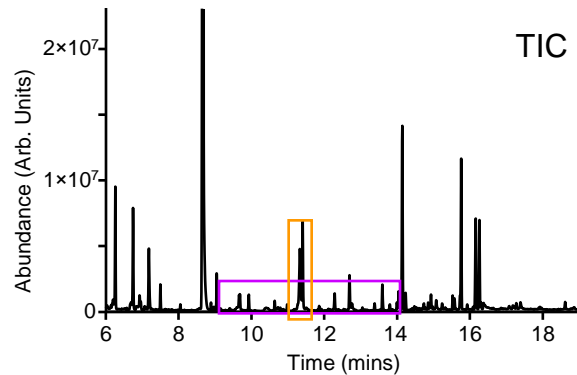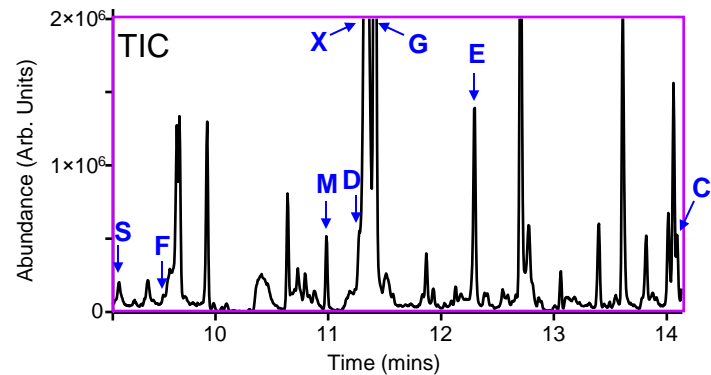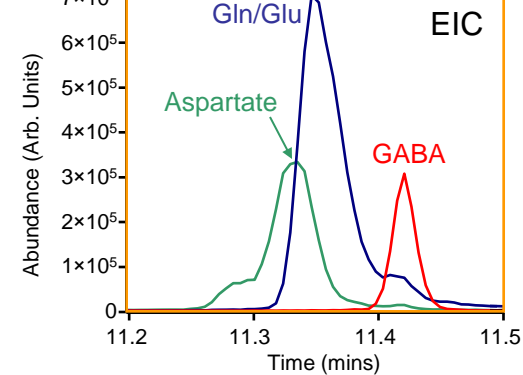

# ASEXUALS

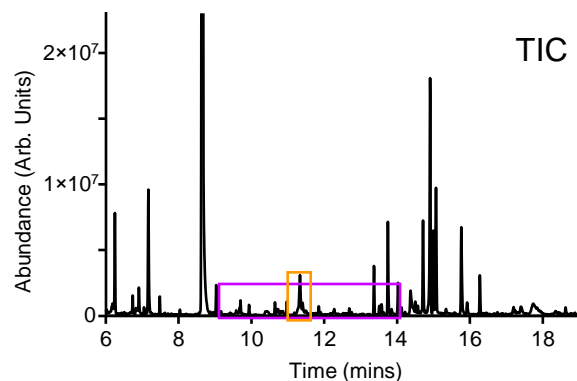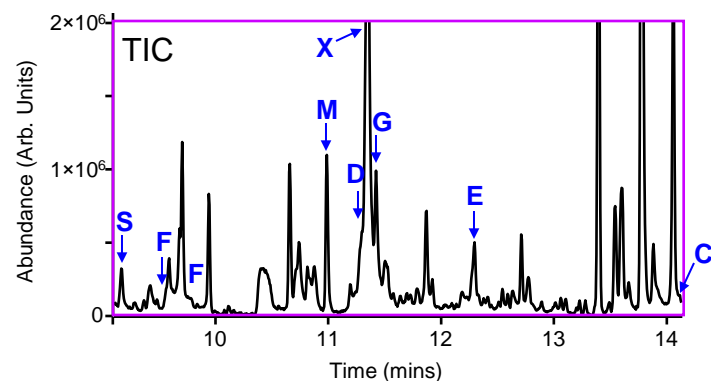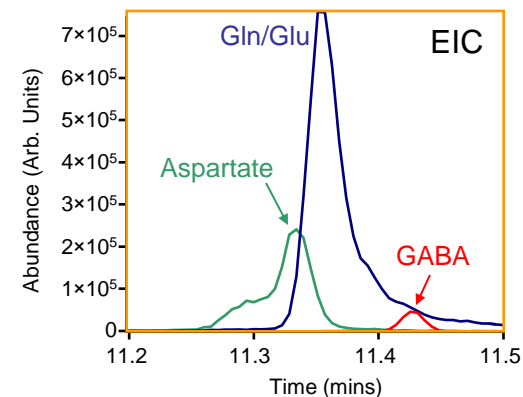

# RBCs

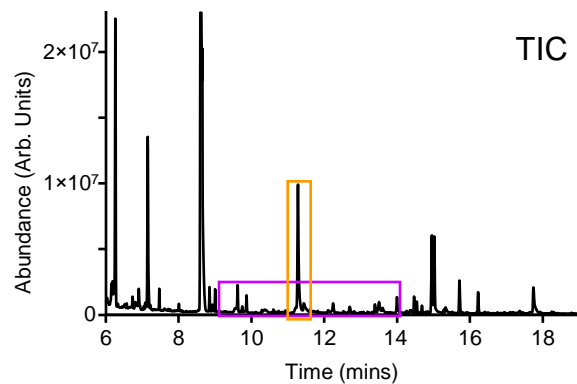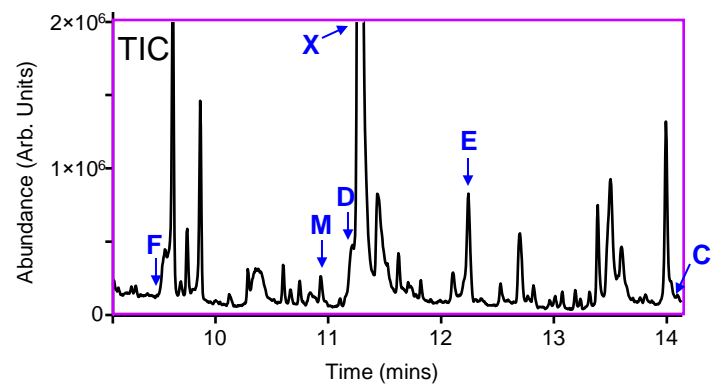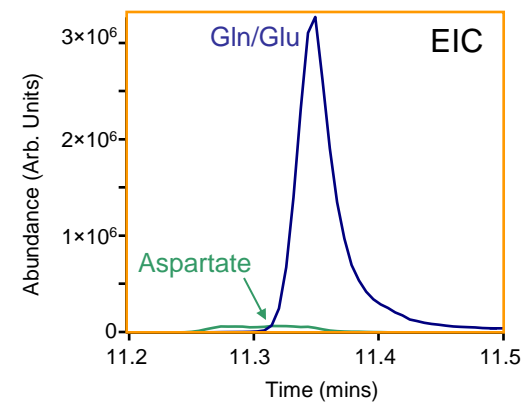

Supplement: Additional file 1 — Gas chromatography–mass spectrometry (GC-MS) chromatograms of Plasmodium falciparum-infected and uninfected red blood cell (RBC) polar metabolites. Gametocytes (top row), schizont-infected RBCs (asexuals, middle row), and uninfected RBCs (bottom row) were harvested, and metabolites extracted as described. The panels depict representative total ion chromatograms (TICs) and extracted ion chromatograms (EICs) of metabolites extracted from 108 cells. Panels in the left-hand column show full TICs. The area of the chromatogram containing the tricarboxylic acid (TCA) metabolites (purple box) is shown in detail in the panels of the middle column. Quantification was performed as described, using extracted ions to distinguish between overlapping peaks. An example is shown in the EIC panels in the right-hand column, depicting the area highlighted by the orange box in the TICs. Lines represent the monoisotopic (diagnostic) ion for aspartic acid (green), glutamine or glutamic acid (blue), and γ-aminobutyric acid (GABA; red) (note the difference in the y-axes). Abundances are shown in arbitrary units, with absolute quantifications shown in Additional file 3. NB: Quantification for glutamic acid was performed using the peak (E) corresponding to glutamate alone. Abbreviations: C, citrate; D, aspartate; E, glutamate; F, fumarate; G, GABA; M, malate; S, succinate; X (Gln/Glu), glutamine/glutamate. [file 1741-7007-11-67-S1.pdf]

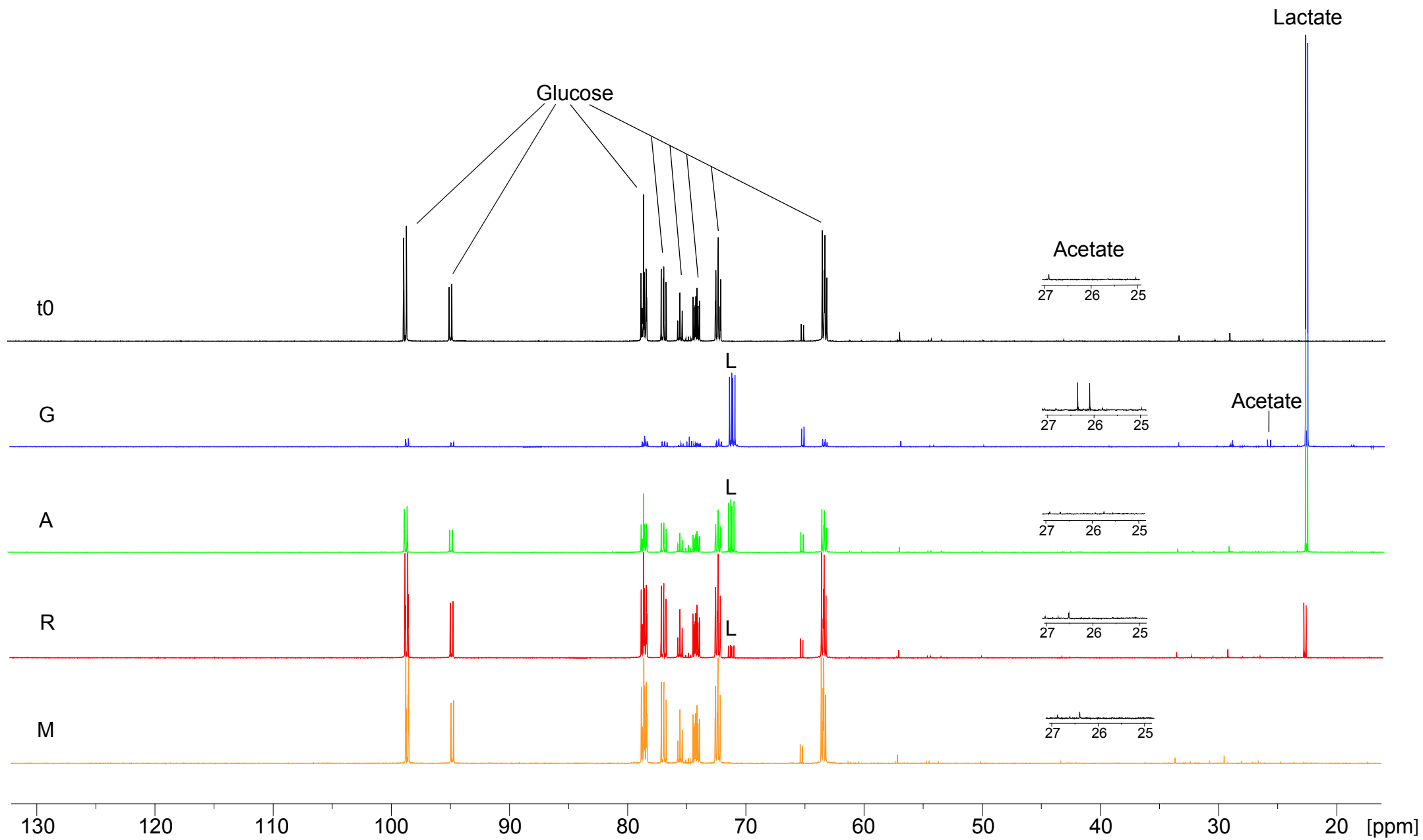

Supplement: Additional file 2 — 13C Nuclear magnetic resonance spectroscopy (NMR) spectra of Plasmodium falciparum-infected and uninfected red blood cell (RBC) culture medium. Early trophozoite-infected RBCs (A), gametocytes (G), and uninfected RBCs (R) were cultivated in medium containing 8 mmol/l 13C-U-glucose. Uptake of 13C-glucose, and secretion of 13C-lactate and 13C-acetate was monitored by 13C-NMR analysis of the medium over 24 hours and compared with the initial medium (t0) and medium lacking RBCs at 24 hours (M). Representative spectra, with peaks corresponding to glucose, lactate, and acetate, are shown. ‘L’ represents the second lactate peak cluster, and a magnified area corresponding to acetate (inset) is shown. Abundances are shown in arbitrary units on an equivalent y-axis, with absolute quantification shown in Figure 2A. [file 1741-7007-11-67-S2.pdf]

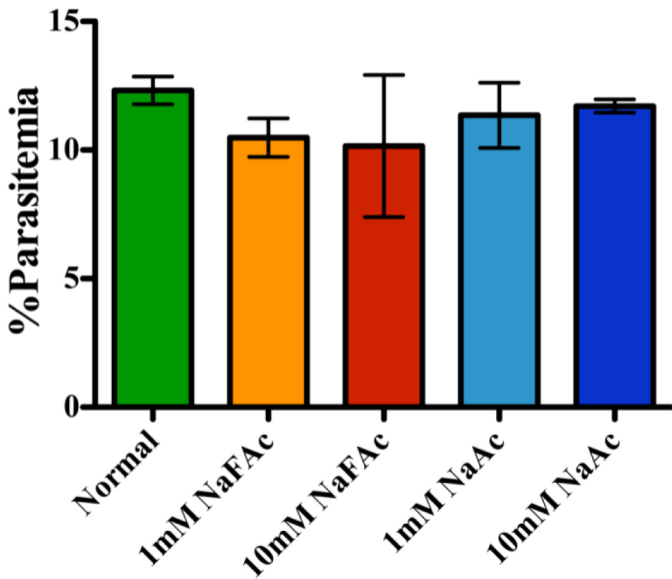

Supplement: Additional file 3 — Quantification of polar metabolites of Plasmodium falciparum-infected and uninfected red blood cells (RBCs) (related to Figure 3A). Ring-infected RBCs, gametocytes, and uninfected RBCs were cultured in the presence or absence of NaFAc, and the metabolic and morphological effects were assessed. (A) After culturing in the presence or absence of 1 mmol sodium fluoracetate (NaFAc), metabolites were extracted from schizont-infected and uninfected RBCs (at 38 hours) and gametocytes (at 24 hours), and polar metabolites of interest were quantified by gas chromatography–mass spectrometry (GC-MS) and comparison with known standards. Abundances are shown in nmoles per 108 cells, with numbers in brackets representing standard deviation, where n = 4 for gametocyte analyses, and n = 3 for asexual stage analyses. The abundance ratio of cells cultured in the presence of NaFAc to the absence of NaFAc is also shown. All values are to 2 d.p. Abbreviations are described in Figures 1 and 3. [file 1741-7007-11-67-S3.pdf]

Control

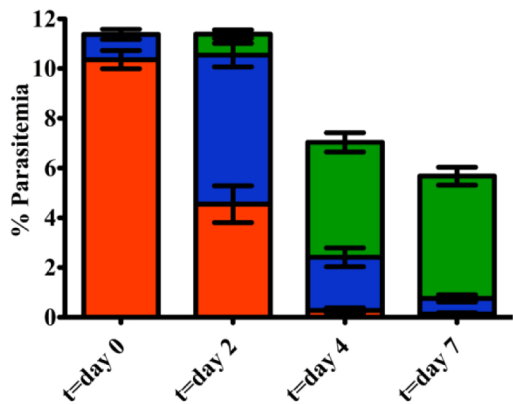

+1mM NaFAc

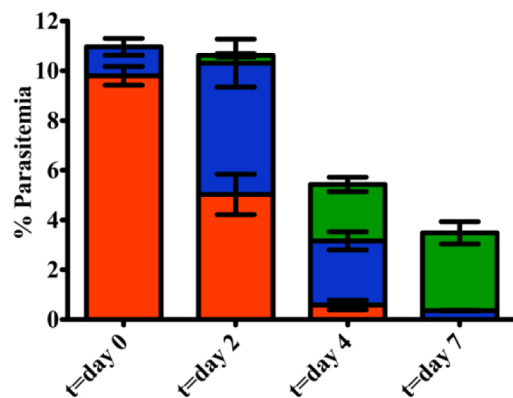

+10mM NaFAc

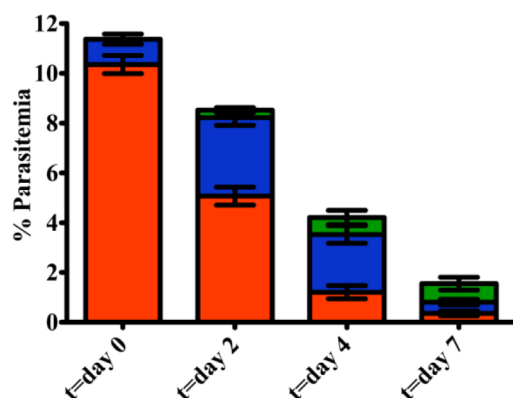

+1mM NaAc

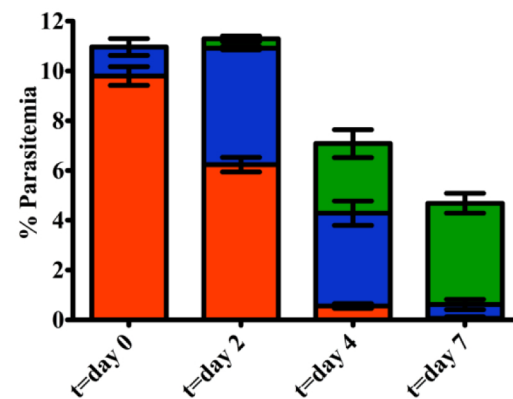

+10mM NaAc

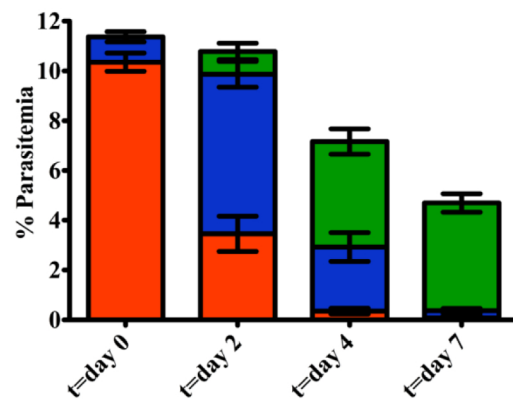

Supplement: Additional file 5 — Growth effects upon treatment of gametocytes with sodium fluoroacetate (NaFAc). A Stage II/III culture of gametocytes was incubated under standard culture conditions with or without the addition of 1 or 10 mmol/l NaFAc or sodium acetate (NaAc). Stage distribution and parasitemia levels were assessed in Giemsa smears made on days 0 to 7 (see Additional file 6). Red, Stage II/III; blue, Stage IV; green, Stage V. Error bars represent SEM where n = 3 biological replicates. [file 1741-7007-11-67-S5.pdf]

Control

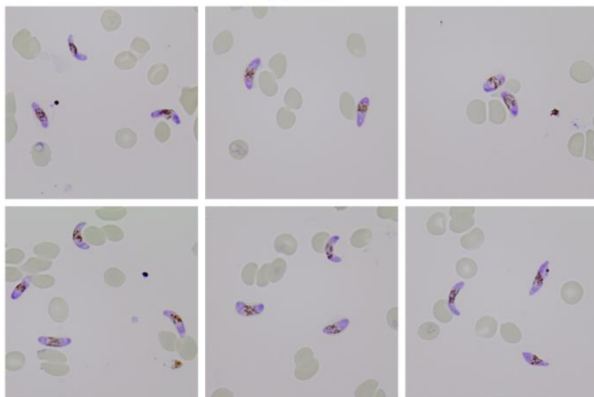

+1mM NaFAC

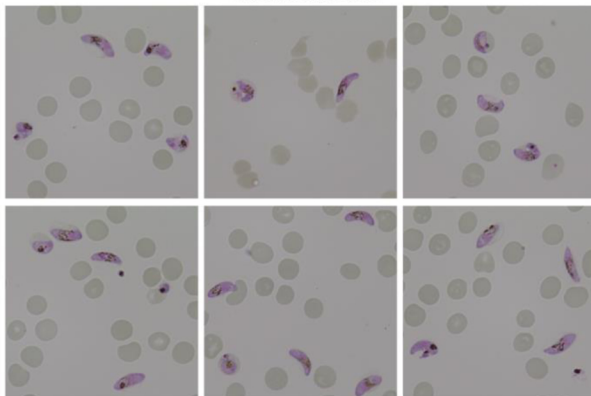

+10mM NaFAC

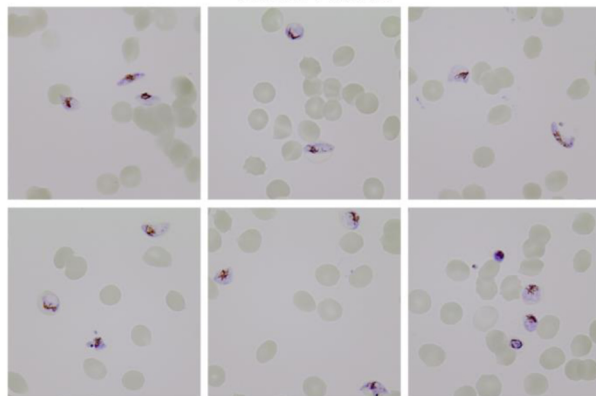

+1mM NaAc

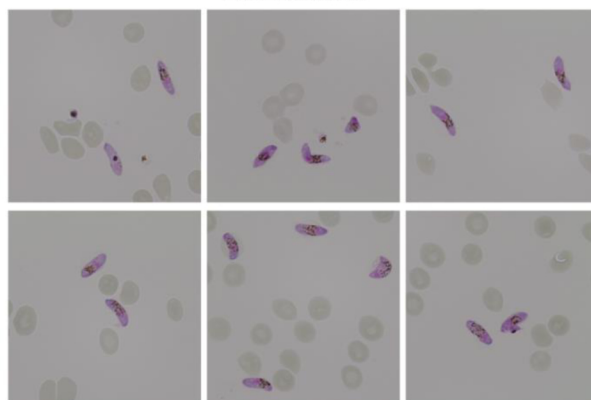

+10mM NaAc

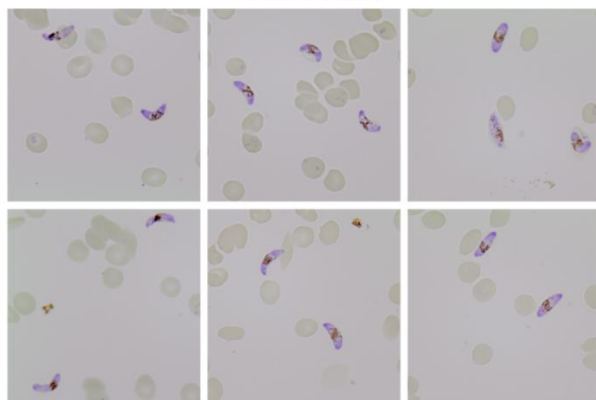

Supplement: Additional file 6 — Giemsa-stained smears of gametocytes with sodium fluoroacetate (NaFAc). A Stage II/III culture of gametocytes was incubated under standard culture conditions with or without the addition of 1 or 10 mmol/l NaFAc or sodium acetate (NaAc). Representative images of smears made on day 7 are presented. [file 1741-7007-11-67-S6.pdf]
